# Comparative Evaluation of Gemini 3.0- and ChatGPT 5.0-Generated Regional Language Informed Consent Forms in Ophthalmology: A Dual-Rater Study in Hindi and Kannada

**DOI:** 10.7759/cureus.105551

**Published:** 2026-03-20

**Authors:** Deepsekhar Das, Bahubali Shetti, Venkatesh Antalmarad, Anshum Choudhary, Kathyayini G Thodupunoori, Sumit Grover, Atindra Narayan

**Affiliations:** 1 Ophthalmology, All India Institute of Medical Sciences, New Delhi, New Delhi, IND; 2 Medicine, All India Institute of Medical Sciences, New Delhi, New Delhi, IND

**Keywords:** ai generated consent, chatgpt 5.0, gemini 3.0, informed consent, large language model, ophthalmology

## Abstract

Purpose: To evaluate the accuracy, linguistic quality, clinical completeness, and real-world applicability of informed consent forms generated in Indian regional languages (Hindi and Kannada) by two large language model-based chatbots for common ophthalmic surgical procedures.

Methods: In this comparative, blinded observational study, two chatbots (Gemini 3.0 (Google, California, USA) and ChatGPT 5.0 (OpenAI, California, USA)) were prompted to generate informed consent documents in Hindi and Kannada for five ophthalmic scenarios: cataract surgery, traumatic corneal perforation repair, therapeutic penetrating keratoplasty, orbitotomy, and squint surgery. Outputs were independently assessed by four ophthalmologist raters (two for each language) using a 10-point scoring system based on correctness, completeness, language and readability, clinical relevance, and real-world applicability. Descriptive statistics were calculated. Paired t-tests were used to compare chatbot performance, effect sizes (Cohen’s d) were estimated, and inter-rater reliability was assessed using intraclass correlation coefficients (ICCs).

Results: Across both languages, Gemini 3.0 demonstrated more consistent performance and higher combined mean scores. In the Hindi cohort, combined mean scores were comparable between Gemini 3.0 (7.85) and ChatGPT 5.0 (8.00), with significant rater-dependent preference variability. In contrast, in the Kannada cohort, Gemini 3.0 significantly outperformed ChatGPT 5.0 (8.8 vs 7.7, p<0.01), with large to extremely large effect sizes (Cohen’s d: 1.23-3.8). Inter-rater reliability was moderate to good for Gemini 3.0 (ICC: 0.62-0.71) and lower for ChatGPT 5.0 (ICC: 0.38-0.59). ChatGPT 5.0 exhibited frequent grammatical and terminological inaccuracies, particularly in Kannada, affecting clinical usability.

Conclusion: Large language models can generate clinically usable informed consent forms in Indian regional languages; however, performance varies significantly between models. Gemini 3.0 demonstrated superior linguistic accuracy, consistency, and clinical suitability. Language-specific validation and mandatory human oversight are essential before clinical implementation.

## Introduction

Informed consent is a cornerstone of ethical and legal medical practice, ensuring that patients understand the nature, benefits, risks, and alternatives of proposed interventions before agreeing to treatment [1]. Inadequate or poorly understood consent can undermine patient autonomy and expose clinicians to medico-legal risk. In countries such as India, linguistic diversity and variable health literacy further complicate the consent process, particularly when standardized documents are unavailable in regional languages.

Ophthalmology involves a wide range of surgical procedures, many of which are elective or semi-elective, making clear communication and shared decision-making especially important. However, clinicians often rely on ad hoc translations or verbal explanations when formal consent documents are not available in the patient’s native language. Such practices may lead to inconsistencies and omissions, reducing the quality and defensibility of consent [2].

Recent advances in large language models (LLMs) have demonstrated their ability to generate coherent and contextually relevant medical text, including patient education material and clinical documentation [3,4]. These tools offer potential advantages in standardising consent forms, reducing clinician workload, and improving accessibility. However, most existing evaluations of LLMs have focused on English-language outputs, with limited evidence regarding their performance in Indian regional languages such as Hindi and Kannada [5,6].

Regional-language medical text generation poses unique challenges, including accurate translation of technical terminology, grammatical correctness, cultural appropriateness, and alignment with local clinical practice. Errors in these domains may impair patient comprehension or compromise the legal validity of consent. Moreover, the subjective nature of language assessment necessitates human evaluation by clinicians familiar with both medical content and local linguistic usage.

The present study aims to comparatively evaluate two extremely popular advanced LLM-based chatbots for their ability to generate informed consent forms in Hindi and Kannada for common ophthalmic surgical procedures. Using a blinded dual-rater design, we assessed accuracy, completeness, linguistic quality, and real-world applicability, with the goal of informing safe and responsible integration of AI-generated consent documents into clinical practice.

## Materials and methods

This was a comparative, cross-sectional evaluation study conducted using anonymised outputs from two large language model-based chatbots, Gemini 3.0 (Google, California, USA) and ChatGPT 5.0 (OpenAI, California, USA). The study was conducted at All India Institute of Medical Sciences (AIIMS), New Delhi, as a one-day observational study. The main objective of the study was to evaluate the accuracy, linguistic quality, clinical completeness, and real-world applicability of informed consent forms generated in Indian regional languages (Hindi and Kannada) by Gemini 3.0 and ChatGPT 5.0 chatbots for common ophthalmic surgical procedures.

Each chatbot was prompted to generate informed consent forms in Hindi and Kannada for five ophthalmic surgical scenarios: phacoemulsification for cataract, traumatic corneal perforation repair, therapeutic penetrating keratoplasty, orbitotomy, and squint surgery. Prompts were standardized across both models to minimise prompt-related bias.

Four independent ophthalmologists (two for Hindi and two for Kannada) with experience in clinical consent and regional language usage served as raters. The raters were blinded to the identity of the chatbots. Each generated consent document was evaluated using a structured 10-point scoring system encompassing correctness of medical content, completeness of information, language and readability, grammatical accuracy, inclusion of relevant and exclusion of irrelevant data, and real-world applicability in the Indian clinical setting.

Scores were assigned independently by each rater for all chatbot outputs in both languages. Descriptive statistics, including mean and standard deviation, were calculated. Paired t-tests were used to compare the performance of Gemini 3.0 and ChatGPT 5.0 separately for each rater and language. Effect sizes were estimated using Cohen’s d to assess the magnitude of differences. Inter-rater reliability was evaluated using the intraclass correlation coefficient (ICC), interpreted according to standard benchmarks. All statistical analyses were conducted using standard statistical software, with a two-sided p value <0.05 considered statistically significant.

## Results

A total of 20 consent documents were evaluated, comprising five surgical scenarios each in Hindi and Kannada generated by two chatbots and assessed independently by two raters. Both chatbots were able to generate complete consent documents across all scenarios; however, notable differences were observed in accuracy, linguistic quality, and real-world applicability.

Hindi-language evaluation

In the Hindi cohort, the combined mean scores across both raters were comparable between the two chatbots, with Gemini 3.0 achieving a mean score of 7.85 and ChatGPT 5.0 achieving 8.00 (Table 1). Despite similar overall performance, rater-specific analyses revealed significant rater disagreements. Rater 1 scored Gemini 3.0 significantly higher than ChatGPT 5.0 (mean difference: +0.8; p=0.016), whereas Rater 2 scored ChatGPT 5.0 significantly higher than Gemini 3.0 (mean difference: -1.1; p=0.0027) (Table 2).

**Table 1:** Hindi-language consent evaluation scores

Hindi consents				
Surgical scenario	Rater 1 (Gemini 3.0)	Rater 1 (ChatGPT 5.0)	Rater 2 (Gemini 3.0)	Rater 2 (ChatGPT 5.0)
Cataract surgery	8.0	7.0	6.5	7.5
Corneal perforation repair	8.0	8.0	7.0	8.5
Therapeutic PK	8.0	7.0	8.0	9.0
Orbitotomy	8.0	7.0	8.0	9.5
Squint surgery	8.0	7.0	9.0	9.5
Mean score	8.0	7.2	7.7	8.8

**Table 2:** Paired-statistical comparison of chatbot performance

Paired-statistical comparison of chatbot performance				
Language	Rater	Mean difference (Gemini 3.0-ChatGPT 5.0)	p value	Interpretation
Hindi	Rater 1	+0.8	0.016	Gemini 3.0 is better
Hindi	Rater 2	-1.1	0.0027	ChatGPT 5.0 is better
Kannada	Rater 1	+1.4	0.001	Gemini 3.0 is better
Kannada	Rater 2	+0.8	0.049	Gemini 3.0 is better

Effect size analysis demonstrated very large to extremely large differences for both raters (Cohen’s d ranging from 1.79 to 2.93), indicating strong rater-dependent preferences rather than marginal differences in output quality (Table 3). Inter-rater reliability was moderate for Gemini 3.0 (ICC=0.62) but low for ChatGPT 5.0 (ICC=0.38), suggesting greater variability and reduced consistency in the evaluation of ChatGPT 5.0’s Hindi outputs.

**Table 3:** Effect size and inter-rater reliability

Effect size and inter-rater reliability					
Language	Chatbot	Cohen’s d	Effect size	ICC	Agreement strength
Hindi	Gemini 3.0	1.79	Very large	0.62	Moderate
Hindi	ChatGPT 5.0	2.93	Extremely large	0.38	Low
Kannada	Gemini 3.0	1.23-3.8	Large-Extremely large	0.71	Good
Kannada	ChatGPT 5.0	1.10	Large	0.59	Moderate

Kannada-language evaluation

In contrast to Hindi, the Kannada cohort demonstrated consistent and statistically significant dominance of Gemini 3.0. The combined mean score for Gemini 3.0 was 8.8 compared to 7.7 for ChatGPT 5.0, yielding a mean difference of +1.1 points in favour of Gemini 3.0 (Table 4). Both raters independently rated Gemini 3.0 significantly higher than ChatGPT 5.0 (Rater 1: p=0.001; Rater 2: p=0.049) (Table 2).

**Table 4:** Kannada-language consent evaluation scores PK: penetrating keratoplasty.

Kannada consents				
Surgical scenario	Rater 1 (Gemini 3.0)	Rater 1 (ChatGPT 5.0)	Rater 2 (Gemini 3.0)	Rater 2 (ChatGPT 5.0)
Cataract surgery	8.5	7.5	5.0	6.0
Corneal perforation repair	8.5	7.0	10.0	8.0
Therapeutic PK	9.0	7.0	9.0	9.0
Orbitotomy	9.0	8.0	10.0	9.0
Squint surgery	9.0	7.5	10.0	8.0
Mean score	8.8	7.4	8.8	8.0

Effect sizes for the Kannada analysis were large to extremely large (Cohen’s d ranging from 1.23 to 3.8), indicating statistically significant differences between the two models (Table 5). Inter-rater reliability was good for Gemini 3.0 (ICC=0.71) and moderate for ChatGPT 5.0 (ICC=0.59), reflecting greater agreement between raters when evaluating Gemini 3.0’s Kannada outputs.

**Table 5:** Common qualitative error patterns observed

Common qualitative error patterns observed		
Domain	Gemini 3.0	ChatGPT 5.0
Medical terminology	Accurate regional terms	Frequent incorrect translations
Grammar	Mostly error-free	Recurrent grammatical errors
Transliteration	Minimal	Incorrect transliteration of English terms
Cultural suitability	High	Variable
Real-world applicability	Consistently suitable	Limited in Kannada
Output consistency	High	Variable

Overall performance across languages

When both languages were considered together, Gemini 3.0 demonstrated a higher overall mean score (8.33) compared to ChatGPT 5.0 (7.85), corresponding to an overall mean difference of +0.48 points (Table 2). Gemini 3.0 also exhibited more stable performance across languages, with consistently higher inter-rater agreement and fewer extreme score variations (Table 3).

Qualitative observations

Qualitative analysis supported the quantitative findings. Gemini 3.0 consistently used appropriate regional medical terminology, demonstrated grammatical correctness, and adhered closely to standard consent structure. In contrast, ChatGPT 5.0 frequently exhibited grammatical errors, incorrect translations of common ophthalmic terms, and inappropriate transliterations, particularly in Kannada, negatively affecting real-world clinical applicability. These qualitative deficiencies aligned with the lower scores, larger variability, and reduced inter-rater reliability observed for ChatGPT 5.0 (Table 5).

Confounding factors

Rater Subjectivity and Linguistic Preference

Although raters were blinded to chatbot identity, individual differences in linguistic sensitivity, familiarity with regional medical terminology, and personal preferences for brevity versus detail may have influenced scoring. This was particularly evident in the Hindi cohort, where rater preferences diverged significantly despite similar objective content quality.

Variation in Clinical Emphasis Between Raters

Raters may have prioritised different aspects of informed consent, such as procedural detail, complication disclosure, prognosis, or post-operative instructions. Such differences can affect overall scores even when the generated content is factually correct, introducing evaluation bias.

Differences in Regional Language Proficiency

Although the raters were proficient in Hindi and Kannada, subtle differences in fluency, dialect familiarity, or exposure to standardized medical Kannada or Hindi could influence judgments regarding grammatical accuracy and real-world applicability.

Non-Standardised Weighting of Evaluation Domains

While a structured scoring framework was used, individual raters may have implicitly weighted certain domains (e.g., correctness or terminology) more heavily than others (e.g., sentence length or style), potentially confounding comparative scores between chatbots.

Prompt Interpretation Variability by Chatbots

Despite using standardized prompts, large language models may interpret prompts differently due to architectural or training differences. Variations in verbosity, structure, or emphasis may reflect prompt-response dynamics rather than inherent model capability.

## Discussion

Artificial intelligence is currently being utilized in a multitude of ways in healthcare. Its use in ophthalmology has seen a tremendous rise in the recent past. They are routinely used in obtaining information from patients as well as healthcare personnel. They are also being used in identifying pathologies based on their clinical pictures, especially fundus images for diseases like diabetic retinopathy, age-related macular degeneration, and glaucoma [7-11]. The authors have identified the limitations of one such popular AI model with respect to cancer patients [12]. Informed consents are extremely vital documents, the construction of which is quite tedious, and the authors have previously studied the role of large language models in the generation of ophthalmology consent forms in English. One of the important findings was that the AI models were in a significant number of situations generating consent with additional relevant information, which was lacking in the standard All India Ophthalmological Society (AIOS) consent forms [6]. As the existing LLMs are capable of generating information in different Indian languages, the authors found it logical to conduct another study testing the more advanced versions of chatbots in generating informed consents in regional languages.

This dual-rater comparative study evaluated the performance of two large language model-based chatbots in generating informed consent documents in Hindi and Kannada for common ophthalmic surgical procedures. The findings demonstrate that while both chatbots were capable of producing structurally coherent consent forms, their linguistic accuracy, clinical reliability, and real-world applicability varied substantially across languages and models.

A key observation was the language-dependent performance of the chatbots. In Hindi, both models achieved comparable overall mean scores, but with pronounced rater-dependent variability. One rater favoured Gemini 3.0 for clarity and consistency, while the other favoured ChatGPT 5.0 for relative completeness. This divergence underscores the subjective component inherent in evaluating patient-facing medical text, even among trained clinicians. Moderate to low inter-rater reliability, particularly for ChatGPT 5.0, suggests that its Hindi outputs were less predictable and more sensitive to individual rater interpretation.

In contrast, the Kannada analysis revealed a consistent and statistically significant superiority of Gemini 3.0 across both raters, with large to extremely large effect sizes. ChatGPT 5.0 demonstrated frequent grammatical errors, incorrect translations of standard ophthalmic terminology, and inappropriate transliteration of English medical terms. Such errors are not merely linguistic but medico-legally relevant, as inaccurate terminology can compromise patient comprehension and potentially invalidate informed consent. These findings highlight the importance of evaluating AI tools in each target language rather than assuming uniform multilingual competence.

The qualitative error analysis further strengthens this conclusion. Gemini 3.0 showed better adherence to established consent structures, more appropriate use of regionally accepted medical vocabulary, and fewer semantic inaccuracies. ChatGPT 5.0, while sometimes more verbose, frequently included incorrect or culturally incongruent terminology, particularly in Kannada. This aligns with prior literature indicating that large language models trained predominantly on English or high-resource languages may underperform in low-resource or morphologically complex languages without targeted fine-tuning [13].

Inter-rater reliability was consistently higher for Gemini 3.0, suggesting greater output stability and predictability, an essential requirement for clinical deployment. Variability in ChatGPT 5.0’s performance raises concerns regarding its unsupervised use in medico-legal documentation. These findings reinforce current ethical recommendations that AI-generated clinical documents should be used only as assistive tools, with mandatory human oversight [3,4].

From a broader perspective, this study contributes to the emerging body of evidence on AI-assisted patient communication in regional languages. Prior studies have primarily focused on English-language outputs or general patient education material [1,2,5]. By specifically evaluating informed consent, a legally sensitive and ethically critical document, this work addresses a significant gap. The results suggest that regional-language validation should be considered a prerequisite before deploying AI systems in multilingual healthcare settings such as India.

Nevertheless, this study had limitations. The sample size was small, restricted to ophthalmology, and involved only two raters for each language. Patient comprehension and acceptability were not assessed. Future studies should include larger numbers of procedures, multiple specialties, more raters, and patient-centred outcome measures. Additionally, longitudinal evaluation is needed to account for model updates over time.

## Conclusions

In conclusion, while large language models show promise in generating informed consent documents in Indian regional languages, performance is highly language- and model-dependent. Gemini 3.0 demonstrated superior linguistic accuracy, consistency, and clinical suitability, particularly in Kannada. Careful validation, language-specific testing, and clinician oversight are essential before clinical adoption.
